# Reliable Quantification of Protein Expression and Cellular Localization in Histological Sections

**DOI:** 10.1371/journal.pone.0100822

**Published:** 2014-07-11

**Authors:** Michaela Schlederer, Kristina M. Mueller, Johannes Haybaeck, Susanne Heider, Nicole Huttary, Margit Rosner, Markus Hengstschläger, Richard Moriggl, Helmut Dolznig, Lukas Kenner

**Affiliations:** 1 Ludwig Boltzmann Institute for Cancer Research (LBI-CR), Vienna, Austria; 2 Institute of Pathology, Medical University of Graz, Graz, Austria; 3 Unit for Translational Methods in Cancer Research University of Veterinary Medicine Vienna (Vetmeduni Vienna), Vienna, Austria; 4 Institute of Clinical Pathology, Medical University of Vienna, Vienna, Austria; 5 Institute of Medical Genetics, Medical University of Vienna, Vienna, Austria; 6 Unit of Pathology of Laboratory Animals, University of Veterinary Medicine Vienna (Vetmeduni Vienna), Vienna, Austria; Inserm, France

## Abstract

In targeted therapy, patient tumors are analyzed for aberrant activations of core cancer pathways, monitored based on biomarker expression, to ensure efficient treatment. Thus, diagnosis and therapeutic decisions are often based on the status of biomarkers determined by immunohistochemistry in combination with other clinical parameters. Standard evaluation of cancer specimen by immunohistochemistry is frequently impeded by its dependence on subjective interpretation, showing considerable intra- and inter-observer variability. To make treatment decisions more reliable, automated image analysis is an attractive possibility to reproducibly quantify biomarker expression in patient tissue samples. We tested whether image analysis could detect subtle differences in protein expression levels. Gene dosage effects generate well-graded expression patterns for most gene-products, which vary by a factor of two between wildtype and haploinsufficient cells lacking one allele. We used conditional mouse models with deletion of the transcription factors *Stat5ab* in the liver as well *Junb* deletion in a T-cell lymphoma model. We quantified the expression of total or activated STAT5AB or JUNB protein in normal (*Stat5ab^+/+^ or JunB^+/+^*), hemizygous (*Stat5ab^+/Δ^* or *JunB^+/Δ^*) or knockout (*Stat5ab^Δ/Δ^* or *JunB^Δ/Δ^*) settings. Image analysis was able to accurately detect hemizygosity at the protein level. Moreover, nuclear signals were distinguished from cytoplasmic expression and translocation of the transcription factors from the cytoplasm to the nucleus was reliably detected and quantified using image analysis. We demonstrate that image analysis supported pathologists to score nuclear STAT5AB expression levels in immunohistologically stained human hepatocellular patient samples and decreased inter-observer variability.

## Introduction

Cancer genome studies revealed insights into molecular pathways, which are driving events for cancer development and progression. Targeting key signaling molecules, which define core cancer pathways, are a central theme in the modern cancer treatment. Some of these drugs are of great success; for example the kinase inhibitor imatinib, which blocks BCR-ABL tyrosine kinase activity in chronic myelogenous leukemia (CML) [1], or the human epidermal growth factor receptor 2 (HER-2) inhibitors used to treat cancer patients, who aberrantly overexpress epidermal growth factor receptor (EGFR) in the tumor cells [2]. Immunohistochemical (IHC) evaluation of deregulated molecules is an integral part in the diagnosis and monitoring of treatment in human cancer. Manual evaluation of immunohistochemically stained cancer specimen is a subjective and highly individual task, which naturally depends on intra- and inter-observer variability. A study on a set of more than 170 observers underscored these interpretation problems, as 24% of estrogen receptor (ER) staining in breast cancer samples were assessed falsely negative [3]. However, as tailored treatment strategies in the clinic depend on judgment of biomarker expression, it is essential to determine exact expression levels in cancer samples and not only to get a qualitative information if a certain biomarker is expressed or not [4], [5]. Beyond that it is of great importance to know which cell type (stromal or tumor cell) shows an enhanced/reduced expression of a certain biomarker.

Targeted treatments become increasingly expensive and thus, correct biomarker quantification is also a critical economic factor. Computer assisted image analysis of IHC stained specimens [6], [7] might be an attractive solution, as studies have shown that under carefully controlled conditions this method is superior to manual assessment [8]–[11]. However, it is not clear yet, if automated image analysis can distinguish subtle differences in protein expression.

Gene dosage, either by the presence of one or two alleles or variation in gene copy number generally affects the corresponding protein levels. For example the human salivary amylase gene (*AMY1*) number, which can vary between populations due to copy number differences, is correlated linearly with Amylase protein amount [12]. Moreover, the protein levels of the cell cycle inhibitor p27KIP1 were exactly half if cells of *p27^Kip1+/−^* mice were compared to their wildtype counterparts [13].

We tested quantitative image analysis tools and evaluated genetically engineered mouse models, which display well-defined and controlled differences of a specific protein levels associated with gene copy number. First, we demonstrate that signal transducer and activator of transcription 5ab (*Stat5ab*) as well as *JunB* hemizygosity in different conditional knockout models translate to 50% of the corresponding protein levels as determined by Western blotting. This confirmed the results obtained from automatic quantitative assessment of STAT5AB and JUNB protein in IHC-stained paraffin embedded sections of the affected mouse tissues. Second, we demonstrate that image analysis can reliably distinguish cancer cells from the tumor stroma and allows individual analysis. Third, we had to rule out that detection and quantification of a nuclear signal e.g. a transcription factor (such as active STAT5 or STAT3) was not altered by the presence of the molecule in the cytoplasm. We used STAT5AB nuclear translocation upon activation in the mouse liver for verification. Fourth, we show that the inter-observer variability judging nuclear STAT5AB of human hepatocellular carcinoma (HCC) patient samples significantly decreased with the support of quantitative image analysis information. Therefore we conclude that image analysis will improve accuracy and reproducibility to score expression levels in immunohistologically stained patient samples.

## Materials and Methods

### Ethics statement

Mice were kept in a specific-pathogen-free facility and all animal experiments were done according to an ethical animal license protocol, approved by the Medical University of Vienna and Austrian Ministry authorities as published [14]. Human liver specimens were obtained from the Medical University of Graz. Tissue samples were registered in the respective biobank and kept anonymous. The research project was authorized by the ethical committees of the Medical University of Graz (Ref. Nr. 20–119 ex 08/09). The study protocol was in accordance with the ethical guidelines of the Helsinki declaration.

### *Alfp-Cre/Stat5ab* mice, *CD4-Cre*/*NPM-ALK*/*JunB* mice, cell lines, and statistical analysis

NPM-ALK transgenic mice were obtained from the lab of Prof. Inghirami [15]. In brief, these mice were generated with an NPM-ALK chimera construct under the control of the murine CD4 promoter. The transgenic cassette (*CD4* cassette) included the minimal CD4 enhancer, the minimal murine CD4 promoter, the transcription initiation site, part of the untranslated first exon and part of the first intron of the murine *CD4* gene but lacked the CD8 silencer. *JunB* conditional knockouts in T-cells of NPM-ALK transgenic animals were obtained by crossing the NPM-ALK transgenes with mice carrying a floxed allele of *JunB* (*JunB^f/f^*) [16]. Specific deletion of JUNB in T-cells was obtained by expression of CRE under the CD4 promoter. The mice developed T-cell lymphomas [17] and analyzed genotypes were NPM-ALK/*JunB^+/+^*, NPM-ALK/*JunB^+/Δ^* and NPM-ALK/*JunB^Δ/Δ^*. Hepatocyte-specific *Stat5ab* hemizygous and homozygous knockout mice (*Stat5ab^+/Δ^* and *Stat5ab^Δ/Δ^*) were generated by crossing conditional *Stat5ab* mice to Alfp-*Cre* transgenic mice [14].

### Immunohistochemistry

Mouse tissues from the above mentioned mice were fixed in 4% paraformaldehyde, embedded in paraffin and 5 µm sections were prepared. The sections of different genotypes were processed on same slides to warrant exactly the same treatment and incubation times. AEC color development was terminated as early as distinct signals were detected on the wildtype sections. For JUNB, phospho-tyrosine 705 -STAT3 (p-STAT3^Tyr705^), STAT5AB and phospho-tyrosine 694 -STAT5AB (p-STAT5AB^Tyr694^) stainings the sections were incubated at 56°C for 2 hours and rehydrated. Steamer pretreatment (95°C for 1 hour) in Tris/EDTA buffer (pH 9, S2367 DAKO) and cooling was done to retrieve antigens for JUNB, p-STAT3^ Tyr705^ and p-STAT5AB^Tyr694^ stainings. For total STAT5AB antigen retrieval an autoclave pretreatment in citrate buffer (pH 6, S2369 DAKO) was performed. The endogenous peroxidase activity was quenched by incubation of the sections in 3% hydrogen peroxide in PBS for 10 minutes. After washing in PBS an avidin and biotin blocking (SP-2001; Vector laboratories) was performed done for 10 minutes. Rabbit polyclonal JUNB (1∶300 dilution, sc-46, Santa Cruz) antibody, rabbit monoclonal p-STAT3^Tyr705^ (1∶80 dilution, #9145, Cell Signaling), rabbit polyclonal STAT5AB (1∶200 dilution, sc-835, Santa Cruz) antibody and rabbit monoclonal p-STAT5AB^Tyr694^ (1∶80 dilution, #9314, Cell Signaling) antibody were used and incubated at 4°C overnight. Afterwards, the slides were washed in PBS and the signal was detected with the IDetect Super Stain System (IDlabs Biotechnology, IDSTM003). Signal was amplified using 3-Amino-9-Ethylcarbazole (AEC, IDlabs Biotechnology, BP1108) under visual control. Formalin fixed and paraffin embedded (FFPE) HCC patient samples were stained for STAT5AB as described above for the mouse samples.

### Data acquisition using the TissueFAXS software

The technology-platform of TissueGnostics GmbH (Vienna, Austria) provides tools to quantify protein expression levels on immunohistochemically (HistoQuest) stained tissue slides or cell preparations. The software programs are based on single cell detection by identification of nuclear structures [6], [18]–[20].

For analysis with the HistoQuest software, hematoxylin staining was used as a master marker for cell identification on the basis of nuclear detection. Furthermore, the average nuclear size, discrimination area, discrimination gray and background threshold for the master marker was specified. The range of intensities of the master marker (hematoxylin) and the immunohistochemical stainings (i.e. AEC signals) were set by autodetection of the software. Regions of interest (ROIs) were defined as indicated, which were analyzed and quantified separately from the surrounding stromal areas. The general setups were done on a representative image. All images were analyzed with the same settings after adjustments. Forward and backward gating was routinely used for quality control of measurements. By clicking on high or low AEC staining intensity cells in the image, the forward gating tool shows the individual staining intensities of selected cells in the scattergram. Backward gating was used to verify data by visual inspection on the original image. The results are visualized in dot plot scattergrams and/or histograms. Cut-offs (to differentiate between positive and negative cells) and gates (to accentuate between cell populations) were set in the dot blots. For statistical analysis the raw data of the analysis were imported into GraphPad Prism, analyzed for significance and processed for data output as whisker box blots. All photomicrographs were taken with same settings (exposure time, signal amplification, objectives). The images of the IHC photomicrographs were taken with a Zeiss Imager Z1 microscope.

### GH-dependent activation of STAT3 and STAT5AB

For the analysis of growth hormone (GH)-dependent activation of STAT3 and STAT5AB in *Stat5ab^+/+^*, *Stat5ab^+/Δ^* and *Stat5ab^Δ/Δ^* mice were injected intraperitoneally with 2 mg/kg recombinant human GH (Immunotools, Friesoythe; Germany) or with saline and sacrificed 30 min thereafter. For subsequent Western blot and immunohistochemical analysis liver tissues were immediately snap-frozen in liquid nitrogen or fixed in 4% phosphate-buffered formalin (Histofix, Lactan, P087.3) for 48 hrs followed by embedding into paraffin using standard procedures as described [21].

### Western blot analysis

Western blotting was done using protein extracts from liver homogenates or lymphoma tissue. Equal amounts of protein lysates (50 µg) were subjected to sodium dodecyl sulphate-polyacrylamide electrophoresis (SDS-PAGE) and then transferred to a nitrocellulose membrane, and blocked in TBS-T/5% BSA for one hour. Blots were incubated with antibodies against JUNB (1∶1000, Santa Cruz Biotechnologies, sc-46), p-STAT3^Tyr705^ (1∶1000 dilution, #9131, Cell Signaling,), p-STAT5^Tyr694^ (1∶1000 dilution, #71-6900, Invitrogen,) and STAT5B (1∶5000 dilution, epitope aa775–788, [14]) overnight at 4°C. Detection was done using electrochemiluminescence detection system (Amersham Biotech). Liver homogenates were prepared using standard methods [22], [23].

## Results

### Determination of the effect of *Stat5ab* and *JunB* hemizygosity on protein levels

**Figure 1:**
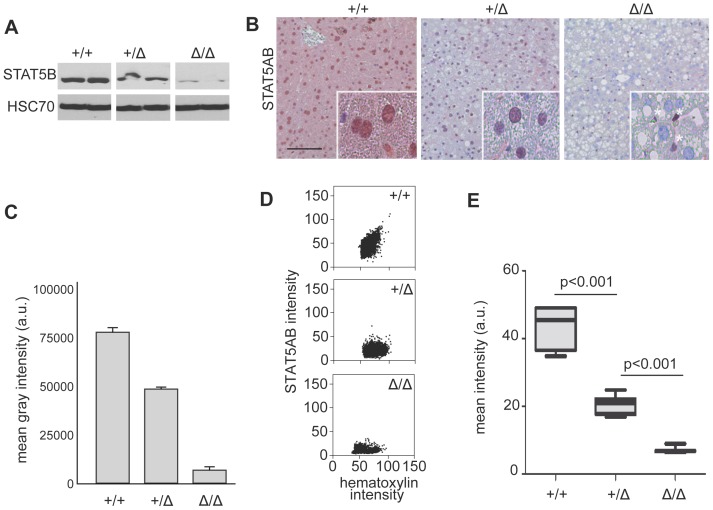
STAT5AB protein expression in mouse Stat5ab^+/+^, Stat5ab^+/Δ^ and Stat5ab^Δ/Δ^ livers. **A** STAT5AB protein expression in *Stat5ab^+/+^* (n = 2), *Stat5ab^+/Δ^* (n = 2) and *Stat5ab^Δ/Δ^* (n = 2) livers determined by Western blot (see Figure S1 in File S1 for non-cropped Western blot) and HSC70 as loading control. **B** IHC staining of STAT5AB in histological sections of the livers (n = 3) used in A. Red, AEC; blue, hematoxylin. Insets are higher magnifications. White stars indicate STAT5AB expression in Kupffer cells and endothelial cells, whereas the hepatocytes are negative for STAT5AB expression. Size bar: 100 µm. **C** Densitometric scanning of the Western blot and quantification of STAT5AB levels (n = 2). Error bars indicate the data range. **D** Dot-blot of STAT5AB IHC quantification from *Stat5ab^+/+^*, *Stat5ab^+/Δ^* and *Stat5ab^Δ/Δ^* livers. **E** Whisker-box blots depicting the quantification of STAT5AB protein levels from D using the HistoQuest software. The box indicates the interquartile range; the horizontal line in the box depicts the median; whiskers indicate the data range. 3788 *Stat5ab^+/+^*, 3041 *Stat5ab^Δ/+^* and 753 *Stat5ab^Δ/^* ^Δ^ cells (hepatocytes) were analyzed in total. One-way ANOVA testing proved that the measured differences were of significance (p<0.001).

In order to evaluate the reliability of automated image analysis and quantification of tissue or cell samples stained with IHC we first used a mouse model, which displays different expression levels of STAT5AB in the liver by conditional deletion of *Stat5ab* [14], [22]–[24]. We demonstrated a gradual decrease in STAT5AB protein amount in livers of wildtype (*Stat5ab^+/+^*), hemizygous (*Stat5ab^+/Δ^*) and homozygous (*Stat5ab^Δ/Δ^*) mice by Western blotting. (Figure 1A and Figure S1 in File S1). These data were consistent with a reduction of STAT5AB immunostaining intensity on liver tissue sections in respective mice (Figure 1B). The remaining low levels of STAT5AB protein in *Stat5ab^Δ/Δ^* cells on Western blots (Figure 1A) could be attributed to STAT5AB positive Kupffer cells and endothelial cells detectable in the IHC (Figure 1B). All hepatocytes were negative for STAT5AB indicating efficient conditional deletion. Quantitative evaluation of the Western blot data (Figure 1C) and automated image analysis of several IHC stains (Figure 1D, E) revealed STAT5AB protein amount in *Stat5ab^+/Δ^* being in the middle between the wildtype and the full knockout. This indicated that the automated image analysis could faithfully distinguish twofold differences in the amount of a specific protein in tissue sections. Our findings on STAT5AB protein expression in *Stat5ab^+/+^*, *Stat5ab^+/Δ^*, *Stat5ab^Δ/Δ^* mice exactly match STAT5AB levels in a different report on *Stat5ab* hemizygosity [25].

To further corroborate our results, we analyzed the expression levels of JUNB protein in experimentally induced mouse nucleophosmin-anaplastic-lymphoma-kinase (NPM-ALK) driven T-cell lymphomas [15] being wildtype, hemizygous or negative for *JunB*. *JunB* deletion was achieved in T-cells facilitating a CD4 driven CRE and a floxed *JunB* construct [17]. The genotypes of the T-cell lymphomas isolated from 10–18 week old mice were *JunB* wildtype (NPM-ALK*/JunB^+/+^*), *JunB* hemizygous (NPM-ALK/*JunB^+/Δ^*) and lacked *JunB* completely (NPM-ALK/*JunB^Δ/Δ^*). JUNB is heterogeneously expressed in tissues due to cell cycle fluctuations of the protein level [21], [26]. First we determined whether the difference in *JunB* gene dosage was directly translated into different protein levels by Western blot analysis (Figure S2A in File S1). Indeed, densitometric quantification revealed highest expression of JUNB in wildtype tumors, whereas *JunB* hemizygosity displayed intermediate expression levels. JUNB expression in knockout tumors was low but detectable, due to about 10–20% of stromal contamination estimated on the basis of histochemically stained tumor sections [15]. After subtraction of the stroma background level of JUNB expression determined in the NPM-ALK/*JunB^Δ/Δ^* tumors, the relative expression levels were approximately two times higher in NPM-ALK/*JunB* wildtype cells compared to the hemizygous *JunB* lymphoma samples (Figure S2A in File S1). IHC on FFPE tissue sections of the same tumor samples revealed equal JUNB expression patterns (Figure S2B in File S1). The intensity of JUNB staining was strong in the *JunB^+/+^*, intermediate in *JunB^+/Δ^* and undetectable in *JunB^Δ/Δ^* lymphomas (except for stromal cells). Due to the cell cycle dependent regulation of JUNB expression, the staining intensity varied between different cells of the same tumors from high to low expression levels to complete absence. Automated image analysis with the HistoQuest software revealed the quantitative differences in JUNB protein expression (scattergrams in Figure S2B in File S1). The mean JUNB intensity (Figure S2C in File S1) in *JunB* wildtype tumors was about twice as high as the levels in *JunB* heterozygote lymphomas, which exactly reflected the results obtained from total protein lysates and Western blots. Therefore, we concluded that controlled IHC staining of different tissues on the same microscopic slide and subsequent automated cell detection based image analysis can reliably distinguish protein level differences of a factor of two.

### Quantification of tyrosine phosphorylated STAT5AB and STAT3^Tyr705^ in hepatic immunostainings match quantitative Western blot data

**Figure 2:**
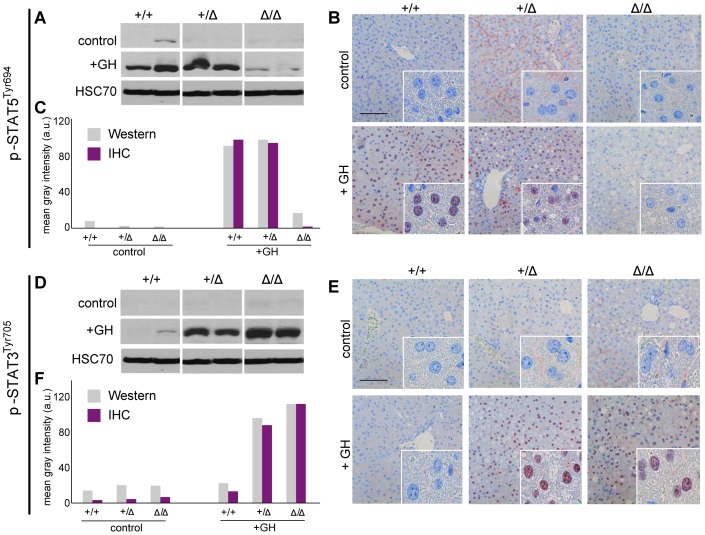
p-STAT5^Tyr694^ and p-STAT3^Tyr705^ expression upon GH treatment compared to control mice. **A** Western Blot of p-STAT5^Tyr694^ expression in *Stat5ab^+/+^* (n = 2), *Stat5ab^+/Δ^* (n = 2) and *Stat5ab^Δ/Δ^* (n = 2) livers of GH treated and control mice. HSC70 is shown as loading control. **B** IHC of p-STAT5^Tyr694^ in sections of the livers (n = 3) used in A. Red, AEC; blue, hematoxylin. Size bar: 100 µm. High magnifications (60x) are shown in insets. **C** Quantification of p-STAT5^Tyr694^ levels from Western blots (grey) by densitometric scanning and IHC (magenta) using the HistoQuest software (*Stat5ab^+/+^*, control; number of cells n = 3276, GH; number of cells n = 2286; *Stat5ab^+/Δ^* control; number of cells n = 2577, GH; number of cells n = 3549; *Stat5ab^Δ/Δ^* control; number of cells n = 3299, GH; number of cells n = 2200). The highest arbitrary expression values from A and B were set to 100 for direct comparison. **D** Western blot of p-STAT3^Tyr705^ expression in *Stat5ab^+/+^* (n = 2), *Stat5ab^+/Δ^* (n = 2) and *Stat5ab^Δ/Δ^* (n = 2) livers of GH treated and control mice. HSC70 is shown as loading control. **E** IHC of p-STAT3 ^Tyr705^ in sections of the livers (n = 3) used in D. Red, AEC; blue, hematoxylin. Size bar: 100 µm. High magnifications are shown in insets (60x). **F** Quantification of p-STAT3^Tyr705^ levels from Western blots (grey) by densitometric scanning and IHC (magenta) using the HistoQuest software (*Stat5ab^+/+^*, control; number of cells n = 2634, GH; number of cells n = 2286; *Stat5ab^+/Δ^* control; number of cells n = 22230, GH n = 2562; *Stat5ab^Δ/Δ^* control; number of cells n = 2414, GH n = 2716). The highest arbitrary expression values from A and B were set to 100 for direct comparison.

Next, we analyzed the activity status via expression of tyrosine phosphorylated (pY) STAT5AB and STAT3**^Tyr705^** in the mouse liver model. The amount of phosphorylated STAT proteins is indicative for their transcriptional activity. STAT5AB phosphorylation in hepatocytes can be achieved by growth hormone administration in mice. As demonstrated earlier, hepatic STAT5AB is heavily phosphorylated upon GH treatment in *Stat5ab^+/+^* mice, whereas the p-STAT5AB^Tyr694^ signal is absent in untreated mice as well as in GH induced *Stat5ab^Δ/Δ^* mice [14], [22]–[24] (Figure 2A). *Stat5ab^+/Δ^* hepatocytes displayed the same level of STAT5AB phosphorylation indicative for the full STAT5AB signaling capacity of hepatocytes containing only half the amount of STAT5AB (Figure 2A). IHC analysis exactly reflected the Western blot data and as expected p-STAT5AB^Tyr694^ was only detected in nuclei (Figure 2B). Unspecific background staining outside the hepatocytes was present in certain areas as depicted in the control *Stat5ab^+/Δ^* liver sections. Convincingly, p-STAT5AB^Tyr694^ levels were almost indistinguishable between quantification obtained from Western blot analysis and automated IHC image acquisition (Figure 2C). Importantly, the background staining in the above-mentioned sections was not incorporated by the analysis software, since only nuclear signals were pre-defined to be valued as specific. Finally we proved that p-STAT3**^Tyr705^** levels were adequately measured by the analysis software and precisely reflect Western blot data (Figure 2D–F). As already described, STAT3 seems to compensate the loss of STAT5AB in liver, and GH can induce STAT3 activity in *Stat5ab^Δ/Δ^* hepatocytes [14], [22]–[24]. This is not the case in *Stat5ab^+/+^* livers, in which no STAT3 activation upon GH occurred (Figure 2D–E). Surprisingly, STAT3 was activated in *Stat5ab^+/Δ^* liver tissue to almost the same extent as in the knockout cells. These findings indicate that the compensatory STAT3 activation is rather induced by low STAT5AB protein levels and not by diminished phosphorylation of STAT5AB.

Taken together we provide a proof of concept study that STAT5AB and STAT3 activity, which can be assessed by the levels of tyrosine phosphorylated protein, can reliably be quantified in FFPE tissue sections using cell based image analysis software.

### Detection and quantification of transcription factor translocation into the nucleus *in vivo* and *in vitro*

**Figure 3:**
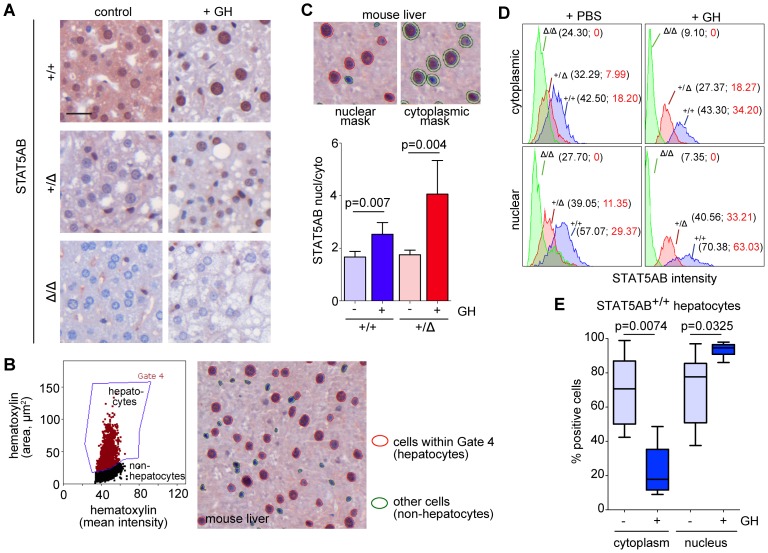
Detection and quantification of transcription factor translocation into the nucleus in mouse livers. **A** IHC of STAT5AB expression in *Stat5ab^+/+^* (n = 3), *Stat5ab^+/Δ^* (n = 3) and *Stat5ab^Δ/Δ^* (n = 3) livers of GH treated and control mice. Size bar: 50 µm. **B** Scattergram of the analyzed IHC samples used in A. Discrimination of hepatocytes due to their hematoxylin area and mean intensity depicted in gate 4. Image of gated (hepatocytes, red circled) and of non-hepatocytic cells, mainly Kupffer cells (green circled) in an IHC stained liver sample. **C** Nuclear and cytoplasmic mask of cells as they are recognized by HistoQuest. The bar graph shows the ratios of nuclear versus cytoplasmic STAT5AB expression levels. Error bars are S.D. Student's t-test was performed to demonstrate statistical significance. **D** Histograms of nuclear/cytoplasmic STAT5AB intensities are shown; *Stat5ab^+/+^* (blue), *Stat5ab^+/Δ^* (red) and *Stat5ab^Δ/Δ^* (green). The corresponding values in the histograms (black values) showed the gradual decrease of the mean intensities. Based on the fact that *Stat5ab^Δ/Δ^* mice have no hepatic STAT5AB their values in the histograms can be set as background intensity. After background subtraction the numbers displayed the expected 2∶1∶0 ratios (red values). **E** Whiskers-box plots depicting the quantification of STAT5AB positive hepatocytes (cytoplasmic C; nuclear N) from *Stat5ab^+/+^* with and without GH stimulation using the HistoQuest software. The box indicates the interquartile range; the horizontal line in the box depicts the median. Whiskers indicate the data range. Student's t-test was used to demonstrate statistical significance.

Next, we wanted to verify that the cell recognition software could consistently distinguish nuclear from cytoplasmic signals. This is important since the activity of certain proteins (i.e. specifically transcription factors) depends on their nuclear localization. Therefore, a proper distinction of nuclear versus cytoplasmic antigen expression is a prerequisite for consistent image analysis and quantification with a specific attribution to the respective cell type that is to be quantified. To test this, we used the GH induction of STAT5AB phosphorylation in the mouse liver, which is accompanied by a translocation of STAT5AB into the nucleus. Nuclear STAT5AB increased in GH treated *Stat5ab^+/+^* and *Stat5ab^+/Δ^* mouse livers as evident by visual inspection whereas the cytoplasmic levels decreased (Figure 3A). After automated nucleus detection, nuclear size was used to discriminate between hepatocytes and stromal cells in the liver and only parenchymal cells were further analyzed (Figure 3B, left, Gate 4). Backgating verified proper gate selection (Figure 3B, right). Ring-shaped virtual masks were generated by software to determine perinuclear staining intensities of STAT5AB in the cytoplasm (Figure 3C).

The mean intensity of nuclear STAT5AB increased while cytoplasmic levels dropped upon GH stimulation in good accordance to the visual impression from the photomicrographs (Figure 3A). This is best visualized by the increase in STAT5AB nuclear versus cytoplasmic ratios in livers of GH treated animals compared to their controls (Figure 3C). The 2∶1∶0 ratio of STAT5AB protein levels in di-allelic (+/+), mono-allelic (+/Δ) or loss (Δ/Δ) of STAT5AB animals remained constant in the cytoplasm and nucleus, both under mock and GH condition as depicted by the respective mean intensities of the histograms (Figure 3D). Based on intensities of background staining in the *Stat5ab^Δ/Δ^* liver cells a cut-off was defined (Figure 3D), which allowed determination of percentages of cells positive or negative for STAT5AB in the nucleus and/or cytoplasm (Figure 3E). There was a significant reduction of cytoplasmic STAT5AB upon GH stimulation accompanied by a significant increase in nuclear STAT5AB.

### Quantification and evaluation of STAT5AB in human hepatocellular carcinoma (HCC) samples

**Figure 4:**
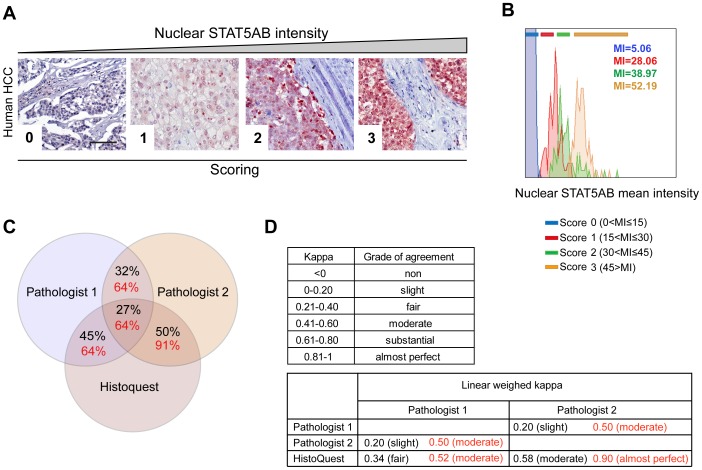
Analysis and evaluation of STAT5AB in human HCC samples and the detection of interobserver variabilties. **A** Representative images of human HCC with different nuclear STAT5AB expression levels (0, 1, 2, 3) which were scored corresponding to pathological evaluation. Size bar: 100 µm. **B** Histograms from image analysis based quantification of nuclear STAT5AB in the representative images from A. Mean intensity (MI) values and scoring zones are depicted. **C** Venn diagram depicting the percent overlaps from two pathologists and image analysis on the assessment of nuclear STAT5AB. **D** Linear weighed kappa analysis depicting the grade of agreement between the evaluating pathologists and the HistoQuest software before (black values) and after software-assisted re-evaluation (red values).

STAT5B activity was shown to be correlated with poor prognosis in patients with HCC [27]. Therefore, we wanted to test whether image analysis could reliably detect differences in nuclear STAT5AB levels in the human samples. Sections from 22 human HCC specimens were stained for STAT5AB and expression intensities of nuclear STAT5AB was determined using the image analysis tool. The nuclear mean intensities were converted into a four level scoring matrix from 0 (no expression) to 3 (strong expression; see Figure 4A, B and Figure S3A in File S1). These samples were independently evaluated in parallel by two board-registered pathologists and judged for nuclear STAT5AB expression based on the same four levels of scoring (Figure S3A, B in File S1). Representative images of the evaluated cancer tissues are depicted in Figure S4 in File S1. The pathologist's grade of agreement on nuclear STAT5AB levels was 32%. Quantitative image analysis showed an accordance of 45% and 50% with the two pathologists, respectively (Figure 4C, Figure S3C in File S1). Determination of linear weighed kappa values to statistically measure the inter-rater agreement [28] revealed only slight agreement between the two pathologists, whereas moderate and fair accordance between the pathologists and automated image analysis (Figure 4D, black values). After one week the pathologists re-evaluated the samples with support of the image analysis scores and the results were analyzed for their percentage agreement and kappa values. The inter-observer variability decreased after providing the software-derived supportive suggestion leading to an increase from 27% to 64% overlap (Figure 4C). Determination of kappa values revealed an increase from slight to moderate accordance between the pathologists and the agreement with the image analysis results raised from fair to moderate and moderate to almost perfect, respectively (Figure 4D, red).

Taken together, we demonstrate that automated image analysis tools in combination with well controlled IHC staining procedures can help evaluating the staining intensity and percentages of positive cells in different sections, providing more objective and comparable results. These techniques can help and support pathologists in quantification of biomarker expression levels, but of course cannot replace a trained pathologists' evaluation of normal or diseased tissues.

## Discussion

One challenge for molecular medicine is to develop standardized staining procedures as well as objective quantification methods for IHC stained tissue samples. The importance of pre-analytical as well as analytical procedures, such as tissue transportation, fixation, sectioning, antigen retrieval and staining procedures is undoubted and extensively discussed [5]. However, accuracy and reproducibility of automated analysis can be obtained only if quantifiable reference standards are available and are employed to normalize the automated system. Standardization of all the assay steps should be performed starting with sample acquisition (fixation and staining) to data report as well as interpretation and analysis (such as accurate and reliable reading score). Here, we focused to optimize the post-analytical process and how to measure and analyze IHC stained patient samples.

It is well documented that different observers see and judge the same tissue section staining differently [29]–[31]. Therefore, one solution to this subjective interpretation is high-end automated image analysis technology [8]–[10], which potentially offer the development of objective scoring models for IHC. Several image analysis software solutions and algorithms have been used [7], [32] and many are commercially available. These tools can also help to generate high throughput analysis in a cost effective manner. It has to be clarified that turnaround time for image analysis if judged individually is longer than pathologist evaluation. However, slides can be scanned automatically in a high throughput manner to speed up the process. In addition, the standardized settings can be used for all samples to be analyzed and guarantee unbiased and reproducible quantitative results. This gets more and more important since there is increased numbers of targeted therapies introduced, for which the patient stratification relies on exact IHC biomarker evaluation of tissue sections.

We worked with a commercially available software (HistoQuest), which uses color separation, as well as cell nuclear recognition algorithms (nuclear segmentation based on hematoxylin staining) to measure IHC staining intensities horseradish peroxidase or alkaline phosphatase based detection using e.g. AEC, 3,3′-diaminobenzidine as chromogenic substrates) in individual cells in the sections. We asked, whether this system is powerful enough to exactly quantify minor expression differences in tissue sections. It is of importance to notice that the tissue specimens were fixed for the same time period, arranged in the same block, cut, stained and analyzed on the same slides, in order to avoid differences in section thickness and staining time. This setup might be of relevance for the acquisition of quantitative data in clinical samples albeit it is difficult to achieve in routine diagnostics. To circumvent the drawbacks of individual preparation and staining of tissue samples, we suggest using an internal control standard, embedded next to the patient sample in the paraffin block to obtain an inter-sample reference for normalization or to use custom made tissue microarrays (TMA) of the samples to be compared.

To obtain defined differences we made use of gene-dosage effects in two independent genetic mouse models, harboring either wildtype *Stat5ab* and *JunB* or the corresponding hemizygous alleles. Full knockouts of these genes were used as negative expression controls. The models offered exact and stable differences of protein expression resulting in relative expression levels of 2, 1 and 0 *in vivo*. The calculated ratio was indeed observed in the analyzed tissue samples, as indicated by the median staining intensities of STAT5AB and JUNB obtained from the automated image analysis. Since the measured expression levels were so close to the theoretical values and the overall protein levels as determined by Western blotting in the test system, we were confident to detect relative subtle differences in expression. The consequence of haploinsufficiency, accompanied by the reduction of protein levels due to loss of one allele, has been underscored in tumor suppressor studies [33], [34]. Many cell cycle inhibitor proteins or human tumor suppressors such as PTEN, p53, BRCA1/2, PML, RB and SOCS proteins are prominent examples, in which gene dosage levels were thoroughly quantified. For example, in *p27^KIP1+/−^* animals the targeted protein was present at exactly half the expression level of wildtype mice as determined by Western blot analysis [13].

In comparison to global analysis of protein expression in tissues by Western blotting, IHC of tissue sections provide major supplemental information. First, one can obtain the distribution of expression in different cell types of the tissue and the spatial variation of expression in a certain cell type. We clearly demonstrated that our approach could distinguish stroma from parenchyma and could provide quantification data of selected tissue substructures. This ensures a more reliable comparison between physiologic or therapeutic states, since the amount of stromal cells can often vary. Second, it is possible to analyze the intracellular localization of protein expression. Image analysis was able to distinguish nuclear versus cytoplasmic expression, calculate mean expression in the two compartments and cytoplasmic to nuclear translocation was also quantifiable. Convincingly, even the 2∶1.0 gene dosage ratio in the genetically different *Stat5ab* mice, was detected in the cytoplasm as well as in the nucleus of mock versus GH treated mice. However, arguably tumor cells might be very different and variable regarding their morphology. This might complicate the use of an automated system. In this respect we are confident that a major advantage of the image analysis used in this study is the segmentation of cells based on nuclear hematoxylin staining. The size and form of a nucleus can be measured and helps to distinguish different cell types within a tissue (e.g. stromal fibroblasts from hepatocytes in HCC). This feature of nuclear recognition might also be exploited to detect and sub-select cell types of same origin but different morphology.

The reliable detection and quantitative analysis of phospho-protein levels in biopsies is essential to identify malfunction of core cellular pathways in cancer [35] and for monitoring the efficacy of targeted therapies [36]. In a defined inducible setting (GH stimulation) *in vivo* we proved that tyrosine phosphorylation of STAT3 and STAT5AB is consistently quantified in IHC-stained mouse liver tissue by the image analysis system. The result of p-STAT3**^Tyr705^** activation upon GH injection of hepatic STAT5AB heterozygous or knockout mice is in line with an important tumor suppressor role of STAT5AB to prevent liver cancer development. STAT5AB protein levels are essential to saturate GH receptor binding sites to prevent mis-activation of p-STAT3**^Tyr705^** by janus kinase 2 (JAK2) in a GH-induced way.

In a pilot study analyzing 22 human hepatocellular carcinoma samples we show that the inter-rater variation in scoring nuclear STAT5AB significantly decreased with the aid of quantitative image analysis information. These results clearly demonstrated that the software–supported histopathological evaluation was beneficial for a more robust and comparable scoring. Detection of nuclear STAT5AB in cellular subpopulations is useful in predicting human liver cancer progression and drug treatment efficacy in the patients as previously demonstrated [27]. Hepatic STAT5 has also been reported to have tumor suppressive function since it regulates hepatoprotective factors, prevents stress kinase activation and has changed detoxification[14], [22], [23], [37]]. Independent of the future outcome on this controversial issue, the determination of nuclear STAT5 levels will be of vital importance for diagnosis and treatment options of HCC.

In the future the ultimate goal of automated analysis of IHC slides should facilitate large-scale, high throughput applications including prognostic marker validation, identification of novel targets and evaluation of targeted treatment efficacy. In general, we need to be aware of the fact that in human disease a lack of quantifiable internal control standards for IHC normalization makes all IHC stainings only to be semiquantitative and a well-trained pathologist is essential for proper diagnosis, since many aspects of histopathology cannot be recognized by a software tool. As we have demonstrated, automated image analysis can detect changes in protein expression in a reproducible manner, however, sometimes the area of expression is more important than the overall expression level. For instance, expression of certain factors at the invasion front of a certain tumor might have more impact than expression in the middle of the same tumor. This is another reason why evaluation by a pathologist is undispensable. However, we want to point out that small staining differences can be made more quantitative and help to reduce inter-pathologist/experimentator interpretation. Of note, we would also like to point out that these image analysis tools might help to better interpret animal experiments for non-pathologists in a preclinical setup.

## Supporting Information

File S1 (PDF)
