# Supplementary material for: Reliable Quantification of Protein Expression and Cellular Localization in Histological Sections
Source: PLoS One. 2014 Jul 11;9(7):e100822. doi: 10.1371/journal.pone.0100822 (PMC4094387; doi:10.1371/journal.pone.0100822)
Supplement: File S1 — (PDF) [file pone.0100822.s001.pdf]

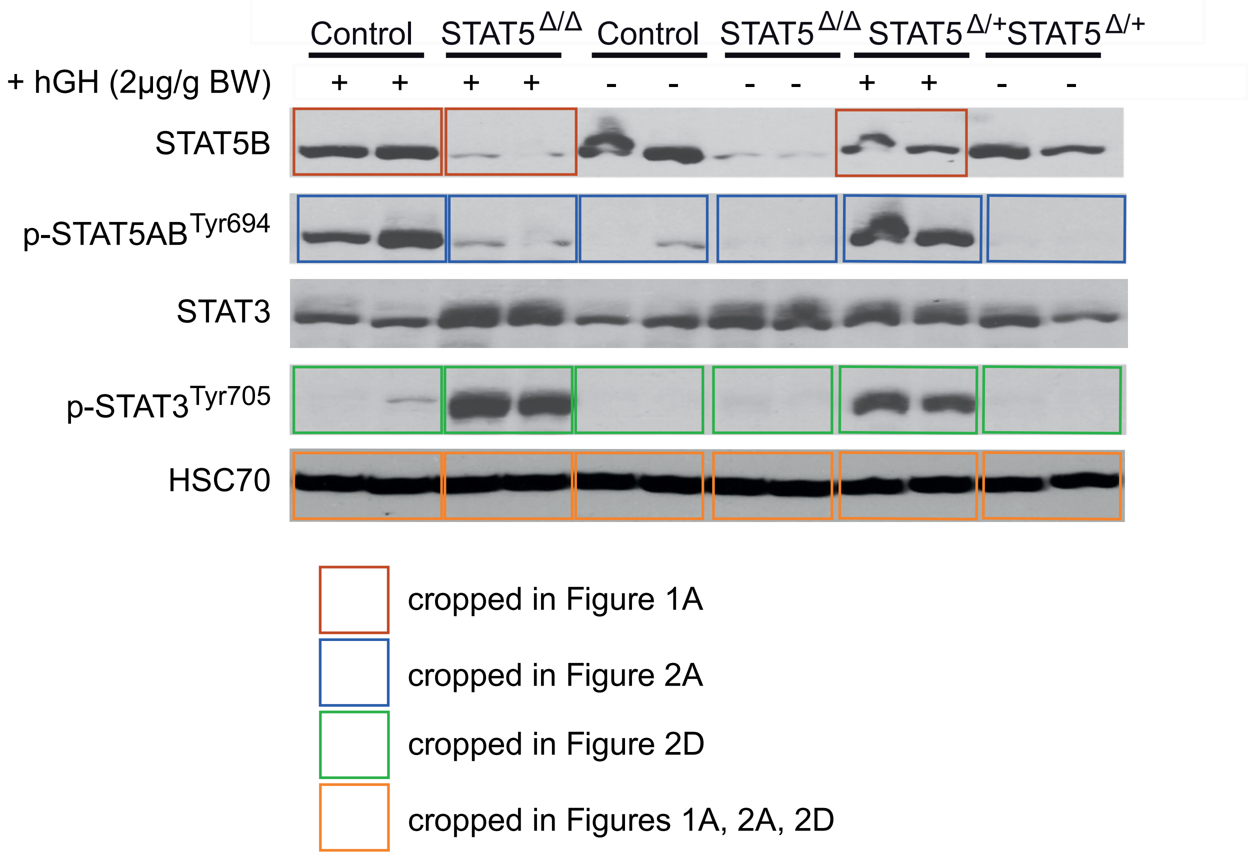

Supplemental Figure S1. *STAT5AB, p-STAT5^{Tyr694} and p-STAT3^{Tyr705} protein expression in Stat5ab$^{+/+}$, Stat5ab$^{+/\Delta}$ and Stat5ab$^{\Delta/\Delta}$ livers with and without GH treatment.* Western blot depicting STAT5AB, p-STAT5^{Tyr694} and p-STAT3^{Tyr705} expression in *Stat5ab$^{+/+}$* (n=2), *Stat5ab$^{+/\Delta}$* (n=2) and *Stat5ab$^{\Delta/\Delta}$* (n=2) livers with and without GH treatment.

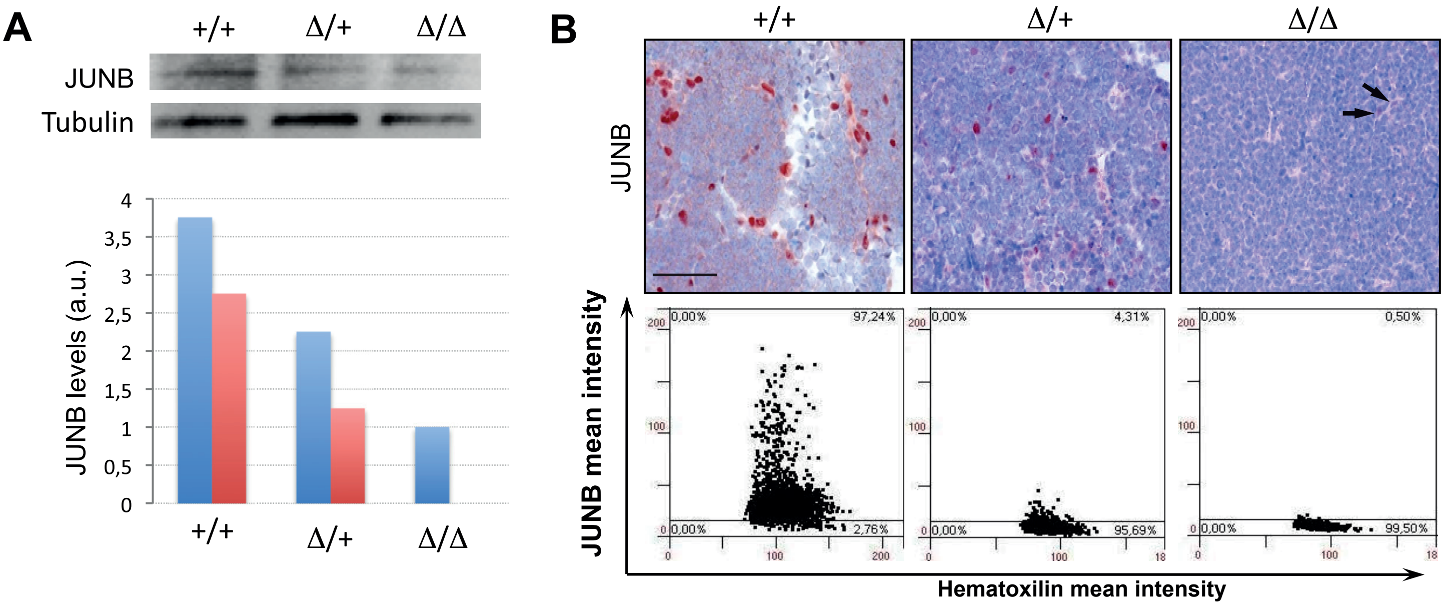

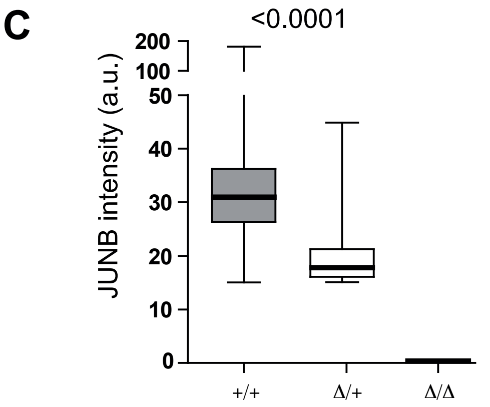

**Total number**	3493	121	11
Minimum	15	15.1	15
Median	31	17.8	16.7
Maximum	181.9	44.8	19.8
Mean	34.7	19.8	16.9
S.D.	18.5	5.2	1.5
Std. Error	0.3	0.5	0.5

Supplemental Figure S2. *Determination of JUNB protein levels in T cell lymphomas.* **A** Western blot of JunB expression in *JunB*$^{+/+}$, *JunB*$^{+/\Delta}$ and *JunB*$^{\Delta/\Delta}$ T-cell lymphomas. **B** T-cell lymphomas stained by IHC for JUNB. JUNB positive cells are stained in red (AEC). Nuclei are counterstained with hematoxylin. Scattergramms show the results from the analysis with HistoQuest™. The background levels were determined in *JunB*$^{\Delta/\Delta}$ tumors and the cut-off was set at 15 mean intensity units. **C** Whisker-box blot from the data generated in (B). The box indicates the interquartile range; the horizontal line in the box depicts the median; whiskers indicate the data range. 3592 *JunB*$^{+/+}$ cells, 2805 *JunB*$^{+/\Delta}$ cells and 2208 *JunB*$^{\Delta/\Delta}$ cells were analyzed in total. One-way ANOVA testing proved that the measured differences were of high significance ($p<0.0001$).

A

Scoring	Mean intensity range
0	(0<MI≤15)
1	(15<MI≤30)
2	(30<MI≤45)
3	(45>MI)

B

	HistoQuest (MI)	HistoQuest (scoring)	Pathologist 1 (first evaluation)	Pathologist 1 (re-evaluation)	Pathologist 2 (first evaluation)	Pathologist 2 (re-evaluation)
1	49.86	3	3	3	2	2
2	33.71	2	3	3	2	2
3	35.42	2	3	3	2	2
4	38.99	2	3	3	2	2
5	28.06	1	2	2	2	2
6	44.79	2	3	2	2	2
7	40.86	2	3	3	2	2
8	35.09	2	3	2	1	2
9	36.50	2	3	3	1	2
10	41.05	2	2	2	1	2
11	44.58	2	3	3	3	2
12	38.97	2	3	2	1	2
13	41.03	2	3	3	1	2
14	62.75	3	3	3	3	3
15	52.19	3	3	3	2	3
16	45.26	3	3	3	2	3
17	34.20	2	2	2	2	2
18	56.24	3	3	3	3	3
19	5.05	0	0	0	0	0
20	52.50	3	3	3	2	3
21	56.19	3	3	3	3	3
22	35.49	2	3	2	2	2

C

**matches**	First evaluation		Re-evaluation	
	Pathologist 1	Pathologist 2	Pathologist 1	Pathologist 2
Pathologist 1		7/22		14/22
Pathologist 2	7/22		14/22	
HistoQuest	10/22	11/22	14/22	20/22

Supplemental Figure S3. *Scoring of nuclear STAT5AB in human HCC samples by two pathologists and the image analysis software.* **A** Matrix for the conversion of the mean intensity (MI) values obtained from image analysis into the scoring values 0, 1, 2 and 3. **B** Evaluation of 22 human HCC samples for nuclear STAT5AB intensity based on the 0, 1, 2, 3 scoring system. The detected nuclear mean intensities (MI) of HistoQuest ™ are listed. The scorings of the two pathologists are displayed before and after software-assisted re-evaluation. Revised scoring values compared to the first evaluation are labeled in red. **C** Numbers of scoring matches in the collective of 22 HCC samples between the pathologists and HistoQuest™ software are depicted.

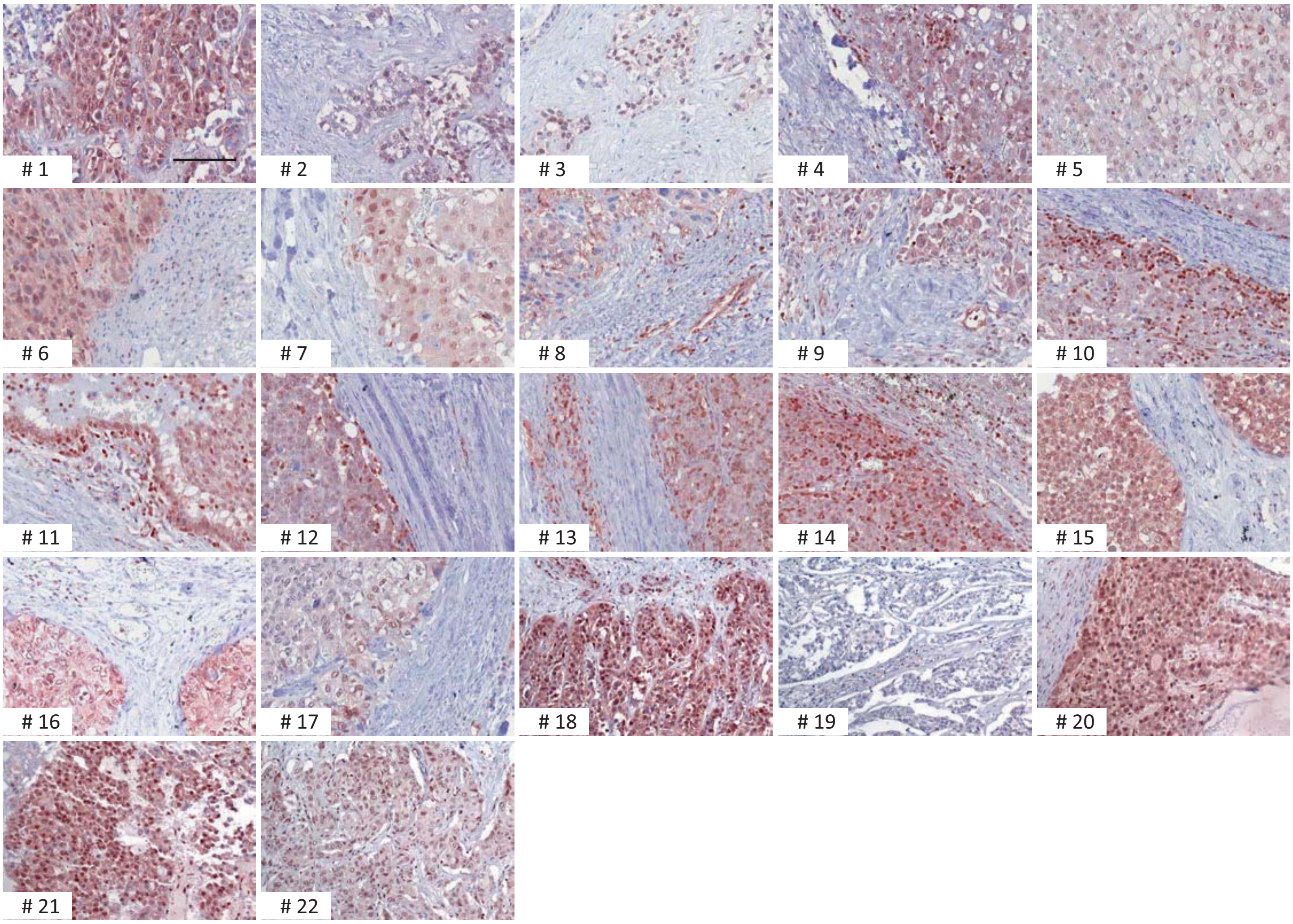

Supplemental Figure S4. *STAT5AB IHC on human HCC specimens used for the evaluation study*. Representative microscopic pictures of 22 human HCC samples, which were stained for STAT5AB expression by IHC to evaluate nuclear STAT5AB levels.
